# Preoperative computed tomography-guided localization of lung nodules with needle placement: a series of cases

**DOI:** 10.1590/0100-6991e-20202890

**Published:** 2021-03-31

**Authors:** RAFAEL EGOROFF FOGOLIN, PAULO CESAR BUFFARA BOSCARDIM, JULIANO MENDES SOUZA

**Affiliations:** 1 - Hospital Nossa Senhora das Graças, Serviço de Cirurgia Torácica - Curitiba - PR - Brasil

**Keywords:** Thoracoscopy, Lung Neoplasms, Tomography Scanners, Image-Guided Biopsy, Videoassisted Thoracic Surgery, Toracoscopia, Neoplasias Pulmonares, Tomógrafos Computadorizados, Biópsia Guiada por Imagem, Cirurgia Torácica Videoassistida

## Abstract

**Objective:**

to report the preoperative localization of pulmonary nodules with the placement of a guidewire oriented by Computed Tomography.

**Methods:**

the nodules were marked using a needle in the shape of a hook or another in the shape of a Q, guided by tomography. The choice of the location for the marking was the shortest distance from the chest wall to the nodule. The marking procedure was performed under local anesthesia and a tomographic control was obtained immediately at the end. Patients were referred to the operating room. Surgical resection occurred less than two hours after the needle placement.

**Results:**

between February 2017 and October 2019, 22 patients aged 43 to 82 years (mean 62.1) were included. The nodules had diameters that varied from 4 to 30 mm and the distance between the nodules and the pleural surface varied from 2 to 43 mm. The location and resection of the nodules were successfully performed in all cases. The guidewire was displaced in five cases. Five patients presented pneumothorax, with the space between the visceral and parietal pleura varying from 2 to 19 mm. In nine patients, an intraparenchymal hematoma of 6 to 35 mm in length was observed without signs, symptoms, or hemodynamic and ventilatory repercussions. The histopathological study was conclusive in all patients.

**Conclusions:**

the localization of pulmonary nodules through guidewires proved to be safe, reliable, and feasible in this series of cases. There was no need for surgical intervention to treat complications.

## RESUMO

**Objetivo:**

relatar a marcação pré-operatória de nódulos pulmonares com o posicionamento de um fio-guia orientado por Tomografia Computadorizada.

**Métodos:**

os nódulos foram marcados utilizando-se agulha em formato de anzol ou outra em formato de Q, orientada por tomografia. A escolha do local para a realização da marcação foi o de menor distância da parede torácica até à lesão. O procedimento de marcação foi realizado sob anestesia local e controle tomográfico foi obtido imediatamente ao término da marcação. Os pacientes foram encaminhados ao centro cirúrgico. A ressecção cirúrgica ocorreu em tempo inferior a duas horas após a marcação.

**Resultados:**

entre fevereiro de 2017 e outubro de 2019, 22 pacientes, com faixa etária entre 43 e 82 anos (média 62,1) foram incluídos. Os nódulos apresentavam diâmetros que variaram de 4 a 30mm e, a distância entre os nódulos e a superfície pleural variou de 2 a 43mm. A localização e a resseção dos nódulos foram realizadas com sucesso em todos os casos. Houve deslocamento do fio-guia em cinco casos. Cinco pacientes apresentaram pneumotóraces, com o espaço entre as pleuras visceral e parietal variando de 2 a 19mm. Em nove pacientes, foi observado hematoma intraparenquimatoso com 6 a 35mm de extensão sem sinais, sintomas ou repercussão hemodinâmica e ventilatória. O estudo histopatológico foi conclusivo em todos os pacientes.

**Conclusões:**

a marcação de nódulos pulmonares por meio de fios marcadores se mostrou segura, confiável e factível nesta série de casos. Não houve necessidade de intervenção para o tratamento de complicações associadas ao método.

## INTRODUCTION

In the last three decades, minimally invasive surgery has been constantly and swiftly developing, greatly changing the surgeon’s routine. The incessant search for diagnostic techniques and more efficient and effective treatment, with fewer complications, lower response to trauma, and ever faster recovery time are the objectives of laparoscopic surgery, which allowed the best surgical results when compared with other techniques^1^.

Thoracic surgery followed this process and today thoracoscopy is a routine procedure, although in Brazil this is not the reality in most services^2^. From more simple procedures, such as pleuroscopy and sympathectomy, to even more complex ones, such as lung lobectomy, bronchoplasty, vascular anastomosis, and radical lymphadenectomy are possible through the minimally invasive approach^3^.

Nodules less than 10 mm in size located more than 10 mm from the pleural surface are a real challenge as for their perioperative locating^4^. In these cases, there is indication of perioperative marking. Several techniques can help the surgeon in the locating process, including spiral markers and contrast media associated with fluoroscopy, cyanoacrylate, methylene blue, ultrasound, and guidewires. The applicability of most of these techniques is quite controversial^5^ ^-^ ^7^. This article reports the experience in the marking of pulmonary nodules with the positioning of a guidewire oriented by Computed Tomography (CT).

## METHODS

**Figure 1:**
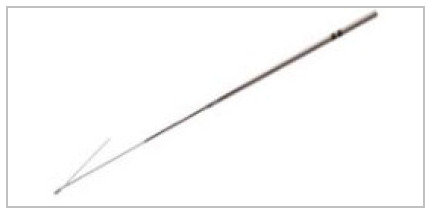
Needle for marking nodules in the hook-shaped - Konpas.

**Figure 2:**
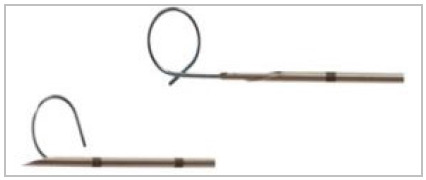
Needle for marking nodules in the Q-shaped - Tuloq.

This is a case series, with the procedures performed in a private, tertiary hospital, from February 2017 to December 2019. The marking of nodules was guided by intermittent CT (Aquilion Multislice 64-channel tomograph, Toshiba Medical, Otawara, Japan), in the hospital’s radiology department, just before surgical resection. We used two guidewires of different configurations, one in the shape of a hook (Figure 1), originally designed to locate breast lesions^8^ and the other in the shape of a “Q” (Figure 2), according to availability at the time of the procedure. Whenever possible, the final portion of the marking guidewire was released shortly proximal to the injury and not inside it, allowing for better anchoring in the tissue. The insertion site was the closest intercostal space, usually the shortest distance to the pulmonary nodule.

The entire procedure was performed under local anesthesia and a control CT was performed immediately after. Patients were operated on less than two hours after the marking. In the Operating Room, all patients underwent general anesthesia and selective intubation with a double-lumen orotracheal tube, and positioned in lateral decubitus. The camera (5mm at 30°), forceps (5mm), and stapler were inserted into the pleural space through two 5 mm and one 12 mm intercostal incisions. During cavity inventory, the marking guidewire was located, and it could even help in the lung tissue exposure for the resection. At the end of the operation, we evaluated the need for chest drainage. The effectiveness of the procedure was assessed by the success in the nodules resection and in the management of complications associated with the marking.

## RESULTS

Between February 2017 and October 2019, 22 patients (10 men and 12 women) of age ranging from 43 to 82 years (mean 62.1), with pulmonary nodules and indication for surgical resection, needed the CT-guided preoperative marking. The nodules diameter varied from 4 mm to 30 mm. The distance between the pulmonary lesions and the nearest pleural surface ranged from 2 mm to 43 mm. The distance traveled by the wire in the lung parenchyma ranged from 7 mm to 62 mm.

**Figure 3:**
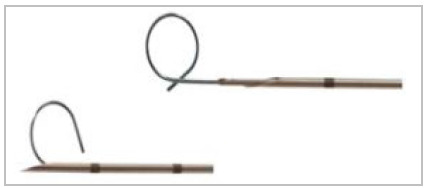
Computed tomography showing the extremity of the needle positioned after the marked nodule.

**Figure 4:**
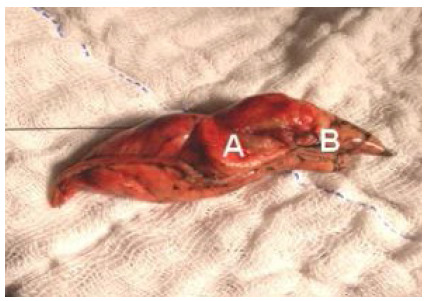
Pulmonary wedge resection demonstrating the positioning of the marking needle.

The nodules occurred in the right upper lobe (5), right lower lobe (7), left upper lobe (4), and left lower lobe (6). The choice of the entry site for the marking was the closest intercostal space, having been anterolateral (2), lateral (7), posterolateral (8), and posterior (5). The locating, marking, and resection of nodes were successful in all cases (Figures 3 and 4).

There was dislodging of the guidewire in five cases, four of them hook-shaped and one, Q-shaped. They were found loose in the pleural space, but it was possible to identify the visceral pleura entry site, allowing locating the lesion, though not ideal. Pneumothorax occurred in five cases (inter-pleural distance 2 mm to 19 mm) and intraparenchymal hematoma, in nine cases (6 mm to 35 mm in diameter), with no signs, symptoms or any hemodynamic or ventilatory repercussions. We did not observe pleuritic pain, air embolism, or hemothorax. The lesions’ histopathological study was conclusive in all cases, which in most part revealed lung adenocarcinoma (13), metastasis from other organs (3), neuroendocrine tumors (1), and benign lesions, such as hamartoma (2), granuloma (2), and anthracofibrosis nodule (1), as recorded in Table 1.

**Table 1:** Characteristics of patients and nodules.

Characteristics	Value (average)
Patients	22
Sex	
Male	10
Female	12
Age	43 a 82 (62,1)
Node size (mm)	4 a 30
Node location	
Right upper lobe	5
Right lower lobe	7
Left upper lobe	4
Left lower lobe	6
Distance from the pleura (mm)	2 a 43 (16)
Distance traveled by the needle (mm)	7 a 62 (29)
Type of needle used	
Hook-shaped	13
Q-shaped	9
Complications	
Pneumothorax	5
Intraparenchymal hematoma	9
Pathological diagnosis	
Lung adenocarcinoma	13
Metastasis	3
Neuroendocrine tumor	1
Hamartoma	2
Granuloma	2
Anthracofibrotic nodule	1

## DISCUSSION

Thoracoscopy allows excellent visualization of the entire outer lung surface, of the chest wall, and of the mediastinum, without the need of rib retractors, decreasing postoperative pain and allowing faster return to daily activities^1^. Most procedures in thoracic surgery can be performed by this minimally invasive technique. However, for resection of pulmonary nodules, location and size can be limiting factors. When the nodules are visible in the collapsed lung or cause retraction of the visceral pleura, there is no need for preoperative marking. Nonetheless, nodules less than 10 mm in size or the ones deep in relation to the pleural surface cannot be located at thoracoscopy^4^.

Nodules with a distance greater than 5 mm from the lung periphery and less than 10 mm in size have a 63% probability of locating failure, with 46% of video-assisted thoracoscopic surgeries needing to be converted to open procedures due to failure in locating the nodule to be resected^9^.

Even nodules with a distance less than 5 mm from the visceral pleura may be difficult to locate, since many display a frosted glass component, with the solid component considerably smaller than the lesion, the latter possibly being in the most distant (proximal) portion of the nodule in relation to the visceral pleura.

The discussion of the image with the radiologist is of utmost importance and should precede the preoperative marking. After observing the percutaneous marking of non-palpable breast tumors, Mack et al. were the first to successfully apply the same technique in lung nodules that would be difficult locate perioperatively. They precisely located nine nodules in six different patients and all were resected with adequate surgical margins and without any complication reported^5^, similar to what we observed in this series.

Injection of methylene blue through the sleeve before placing the guidewire has been reported with the goal to ensure locating the nodule if the wire detaches from the lung^5^ ^-^ ^12^. However, this can quickly dye the lung tissue, interfering with the identification of the lesion site.

Five patients of this series (22.75%) showed asymptomatic pneumothorax, corroborating the data of a retrospective study with 181 patients who had the pulmonary nodules labeled with hook-shaped wires, asymptomatic pneumothorax affecting 30% of them. Still in the same study, intraparenchymal bleeding occurred in 36% of patients, versus 40.9% in our series. The probability of success of the nodule resection in that study was 95%^10^, while in our series it was 100%.

The Q-shaped or the lung specific, spiral shape wires provide more effective marking in relation to the hook-shaped one, displaying a lower risk of displacement from the lung, as was the case with four patients. There is a limitation to the use of those wires due to their very high cost when compared with the hook-shaped one; they are usually not covered by health insurance carriers or by the public health system. It is believed that the marking wire is best positioned when released immediately proximal to the lesion, so that if it is pulled, the hook will anchor in the nodule instead of away from it^11^. A previous study has reported pleuritic pain in some of the patients and chest wall hematoma^12^. In this series, there was no such event and we observed no other complications.

A comparison between the hook-shaped wire and the methylene blue reported equivalent success rates in locating the nodules (100%) and total complications (54% vs. 46%, p = 0.45). The most frequent complications were pneumothorax and intraparenchymal hemorrhage, and wire detachment took place in 13% of patients, which did not prevent nodule resection. Nonetheless, in that study two patients in the wire marking group had serious complications that prevented further surgery. One had air embolism and the other, electrocardiographic alterations^13^. In the absence of specific, Q-shaped lung marking wires, the use of methylene blue can be considered the economically viable alternative, with good results.

New techniques for nodules marking are under development, with the use of hybrid rooms and new technologies. The location of nodules by electromagnetic navigation seems promising; however, initial results showed a 94% success rate in locating the lesions and a 7% rate of conversion to thoracotomy^14^.

## CONCLUSIONS

The preoperative marking of small pulmonary nodules or those deep in relation to the visceral pleura by guidewires was safe, reliable, and easy to perform. In this series, the Q-shaped wires detached fewer times from the lung parenchyma when compared with the hook-shaped ones. Further studies with marking equipment specific for pulmonary tissue are needed to prove its real effectiveness.
